# High-selectivity profiling of released and labeled N-glycans via polar-embedded reversed-phase chromatography

**DOI:** 10.1007/s00216-018-1495-7

**Published:** 2018-11-26

**Authors:** Johannes G. Wilhelm, Marco Dehling, Fabian Higel

**Affiliations:** 1 Technical Research and Development, Global Drug Development Novartis, Hexal AG, Keltenring 1+3, 82041 Oberhaching, Germany; 2 Present Address: Physico-Chemical Analytics, Protein Sciences, MorphoSys AG, Semmelweisstr. 7, 82152 Planegg, Germany; 3 Present Address: Analytical Development Biologicals, Boehringer Ingelheim Pharma GmbH & Co. KG, Birkendorferstr. 65, 88397 Biberach an der Riss, Germany

**Keywords:** Glycoprotein, N-Glycosylation, UHPLC, Reversed phase, Mass spectrometry

## Abstract

N-Glycosylation is the most complex post-translational modification of proteins and involved in many physiological processes and is therefore of major interest in academic research and in the biopharmaceutical industry. Reliable, robust, reproducible, and selective analysis of N-glycans is essential to understand the multitude of biological roles of N-glycosylation. So far, hydrophilic interaction liquid chromatography analysis of 2-AB or 2-AA derivatized N-glycans has been the standard method. In this work, the superiority of reversed-phase chromatography for complex N-glycosylation analysis is demonstrated. Separation of N-glycans derivatized with anthranilic acid on polar-embedded stationary alkyl phases with sub-2-μm particles results in outstanding selectivity and resolution. In combination with the highly mass spectrometry–compatible mobile phase, even very complex glycan mixtures can be separated, identified, and quantified precisely and accurately. The presented methodology can be applied broadly from basic research to analytical control and release testing of biological drug products and can be implemented in analytical laboratories with minimal effort.

**Electronic supplementary material:**

The online version of this article (10.1007/s00216-018-1495-7) contains supplementary material, which is available to authorized users.

**Graphical abstract:**
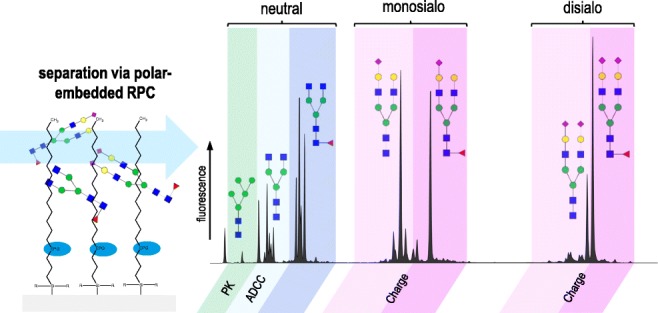
ᅟ

## Introduction

N-Glycosylation is a post-translational modification of asparagine side chains. It is by far the most complex known modification of proteins and is involved in many physiological processes [1]. In the biopharmaceutical industry, N-glycan analysis is of high importance during development and for the control and release of biological drug products [2, 3]. Diversity of N-glycosylation arises from the intricate dynamics of glycan biosynthesis and processing, involving numerous enzymes, which are influenced by changes in cell culture environment, such as pH, temperature, and nutrient concentration. Additionally, N-glycans display a high grade of microheterogeneity by virtue of the multitude of available monosaccharides and their linking glycosidic bonds and branching. In consequence, the analysis of these diverse structures is still a great challenge, despite the availability of commercial kits and the abundance of analytical techniques which have been applied to this task. With the increasing number of complex glycosylated non-IgG and new format biopharmaceuticals in development and the knowledge about the importance of N-glycosylation in physiologic processes as well as their involvement in the development of diseases, there is a high demand for sensitive analytical methods with high resolving power.

N-Glycans can be analyzed on the level of the intact protein, glycopeptides, or released N-glycans [4]. In the biopharmaceutical industry, the analysis of released N-glycans is the method of choice providing the highest sensitivity. For almost a decade, hydrophilic interaction liquid chromatography (HILIC) in combination with fluorescence detection via fluorophore labeling, for example using 2-aminobenzamide (2-AB) or anthranilic acid (2-AA), has been the method of choice for N-glycan analysis [5–7]. Labeling through reductive amination is a fundamental requirement to detect N-glycans via UV or fluorescence, since carbohydrates do not have sufficient UV absorbance to be suitable for UV detection [5]. There is a variety of different labels available on the market with several newly introduced labels, mainly developed to enhance ionization efficiency to improve mass spectrometric analysis [8, 9]. Some companies introduced rapid tagging reagents, usually containing an N-hydroxysuccinimidyl carbamate group which reacts with the terminal amine of glycosylamine-carrying released glycans, such as the RapiFluor-MS tag described by Lauber and colleagues [10]. However, separation of these “click-chemistry” labels is not advantageous over classic labels and reductive amination is still more commonly applied and offers flexibility and independence from specific kits.

Multiple manufacturers of chromatography columns have developed specialized stationary phases for HILIC, which offer high selectivity for different glycan species. Retention on HILIC phases separates N-glycans according to hydrophilicity of the analyte which increases with the size of N-glycans. Alternative approaches utilizing reversed-phase chromatography (RPC) or porous graphitized carbon (PGC) have been developed and reported [11–14]. Analysis with RPC results in separation of labeled N-glycans according to the N-glycan-type complex, hybrid or high mannose. However, presented methods lack complete separation of N-glycan groups [12, 13]. When using RPC for N-glycan analysis, 2-AA has been proven to be advantageous [13]. Thus far, RPC separations were performed almost exclusively on octadecylsilane (ODS) phases and most published approaches are nanoLC-MS based. Most RPC applications were developed for antibodies with a rather simple N-glycosylation pattern and suffer from excessive run times and low resolution, as noted by Vreeker and Wuhrer [15].

In recent years, advances have been seen in the production and packing of new stationary phases for ultra-high-performance chromatographic analysis, such as highly efficient sub-2-μm particles and the advent of alkyl-ligated stationary phases with embedded polar groups (EPG) such as carbamate and amide groups. It was recognized that these phases present unique selectivity for polar analytes [16, 17].

In this work, a novel analytical method for the analysis of complex mixtures of 2-AA-labeled N-glycans on alkyl-ligated stationary phases with embedded amide functionality is presented. It is demonstrated in a systematic approach that the use of this type of stationary phase provides increased selectivity compared to HILIC or conventional ODS phases. In addition, all parts of the analysis including sample preparation, fluorescence detection, and mobile phase composition were optimized. The presented method can be implemented with minimal effort in analytical laboratories and represents a robust and orthogonal alternative to HILIC for N-glycosylation profiling.

## Materials and methods

Rapid PNGase F was purchased from New England BioLabs (Frankfurt am Main, Germany). Acetonitrile, 1-butanol, ethanol, acetic acid, 1-propanol, and 96-well filter plates were obtained from Merck (Darmstadt, Germany). Formic acid, 2-methylpyridine borane complex, and 2-anthranilic acid were acquired from Sigma-Aldrich (Munich, Germany); 96-well Strata SI-1 SPE plates were from Phenomenex (Aschaffenburg, Germany). Monoclonal antibodies and Fc-fusion proteins were procured from in-house development at Novartis. Reversed-phase chromatographic columns were from Phenomenex (Luna Omega 1.6 μm C18; 150 × 2.1 mm; Luna Omega Polar 1.6 μm C18; 150 × 2.1 mm) and Agilent (Zorbax RRHD Bonus RP 1.8 μm C14; 150 × 2.1 mm) and the HILIC column was purchased from Waters (ACQUITY UPLC Glycan BEH Amide 1.7 μm, 150 × 2.1 mm).

### Sample preparation

Sample preparation is based on 96-well plates and thus amendable for automation and high throughput. Samples of low concentration (< 5 mg/mL) were concentrated in 96-well filter plates. Buffer exchange was carried out for sample matrices that caused interference in fluorescence detection; 50–150 μg buffer-exchanged glycoprotein in 10 μL volume was either heat denatured at 95 °C for 5 min (mAbs) or denatured with 10 μL 30 mg/mL RapiGest Surfactant (Waters, Milford, USA) at 95 °C for 10 min (complex biopharmaceuticals). After denaturation, samples were cooled down to ambient temperature. Ten microliters PBS containing 1.5 μL Rapid PNGase F was added per sample. De-N-glycosylation was carried out at 50 °C for 30 min. Samples were cooled to room temperature, and subsequently, 40 μL labeling solution (0.73 M 2-anthranilic acid or 2-aminobenzamide, 0.75 M 2-methylpyridine borane complex in 85:15 ethanol:acetic acid mixture) was added to the released N-glycans without prior purification. Labeling was done at 65 °C for 2 h. For the removal of the reaction mixture, the solution was evaporated to dryness in a vacuum concentrator (Martin Christ, Osterode am Harz, Germany). Glycans were reconstituted in 40 μL of ultrapure water; 35 μL of the resulting solution was diluted with 400 μL acetonitrile in a separate well. The removal of excess label and by-products of the reductive amination was facilitated with the use of Strata SI-1 SPE plates. Liquid was passed through the wells by centrifugation. Plates were pre-conditioned with water and acetonitrile. The diluted sample was then passed through the SPE columns for loading. The column was then washed three times with 1 mL 96% (*v*/*v*) acetonitrile containing 2% (*v*/*v*) formic acid for removal of excess label and other reaction products. Captured N-glycans were eluted with 150 μL of ultrapure water, which was passed through each column twice.

### UHP-RPC with fluorescence detection

Chromatographic separation was performed on a 1290 Infinity II UHPLC system (Agilent, Waldbronn, Germany) optimized for ultra-low dispersion, operated at a flow rate of 0.250 mL min^−1^ and a temperature of 70 °C. Fluorescence detection of 2-AA-labeled N-glycans was carried out with an excitation wavelength of 350 nm and emission wavelength of 440 nm. Fluorescence detection of 2-AB-labeled N-glycans was carried out with an excitation wavelength of 250 nm and emission wavelength of 425 nm. Mobile phase A was composed of H_2_O with 0.5% acetic acid and 0.5% formic acid and mobile phase B of 20% acetonitrile, 5% 1-propanol, 5% 1-butanol, 0.5% acetic acid, and 0.5% formic acid. For complex glycan mixtures, the applied linear gradient started at 2% B and increased to 35% B at 33 min, then increased to 95% B until 48 min and held for 5 min. Subsequently, the column was equilibrated at starting conditions for 5 min. For glycan mapping of mAbs, the linear gradient started at 2% B; the volume fraction of mobile phase B was then increased to 21% at 19 min, then increased to 85% B at 24 min and held for 2.5 min. The column was finally equilibrated at starting conditions for 3.5 min.

### UHP-HILIC with fluorescence detection

HILIC separation of glycans was performed on a 1290 Infinity II UHPLC system (Agilent, Waldbronn, Germany) optimized for ultra-low dispersion, operated at a flow rate of 0.6 mL min^−1^ and a temperature of 55 °C. Fluorescence detection of 2-AA-labeled N-glycans was carried out with an excitation wavelength of 350 nm and emission wavelength of 440 nm. Fluorescence detection of 2-AB-labeled N-glycans was carried out with an excitation wavelength of 250 nm and emission wavelength of 425 nm. Mobile phase A was composed of pure acetonitrile and mobile phase B of 100 mM ammonium formate at pH 4.5. Complex glycan samples were separated with a linear 55-min gradient starting at 26.5% B, which was increased to 45% B. Column wash was performed at 0.5 mL min^−1^ and 70% B for 1 min. Separation of mAb N-glycans was performed with a shorter gradient, increasing from 26.5% B to 38% B over 20 min. Subsequently, the flow was increased to starting conditions over 0.7 min and the column was equilibrated for 2.7 min.

### Assessing the degree of deglycosylation by reducing CGE

Completeness of de-N-glycosylation was assessed using reducing SDS-CGE. After incubation with PNGase F, samples were reduced and denatured. Samples were therefore mixed with 65 μL CE buffer (1% SDS in 50 mM Tris, pH 9) and reduced with 5 μL β-mercaptoethanol at 70 °C for 10 min. Reducing CE-SDS analysis was conducted with the PA 800 Plus (Beckmann-Coulter) capillary electrophoresis system. Samples were injected electrokinetically at 3 kV for 20 s and separated at 12 kV with a run time of 30 min. The UV absorbance signal was collected with 2 Hz at 214 nm.

### Determination of selectivity and peak capacity

Selectivity was determined by plotting the retention time of two tested conditions (e.g., column or mobile phase). Linear regression was then performed and the correlation coefficient *r*^2^ was determined. Overall selectivity was calculated as described by Neue et al. with the following equation [16]:

1 $$ {s}^2=1-{r}^2 $$

where *s*^2^ being the selectivity difference and *r*^2^ the correlation coefficients. An *s*^2^ value of 0 indicates that there is no selectivity differences whereas an *s*^2^ value of 1 means that the compared methods are orthogonal. Conditional peak capacity was determined using the gradient time between the first (*t*_R1_) and last eluting peak (*t*_Rn_) and the average peak width *w*, as described by Neue et al. [18]:

2 $$ {P}_{\mathrm{c}}=1+\left(\left[{t}_{\mathrm{R}\mathrm{n}}-{t}_{\mathrm{R}1}\right]/w\right) $$

### MS identification of 2-AA N-glycans

To identify 2-AA N-glycans based on their exact mass, a post-column splitter (Analytical Scientific Instruments, CA, USA) was used to split the flow after the column in a ratio of approximately 1:2 to the fluorescence detector and a Q Exactive Plus mass spectrometer (Thermo Fisher Scientific, Germering, Germany) operated under Chromeleon 7.2. The MS was operated with positive polarity with a spray voltage of 3.5 kV, a capillary temperature of 275 °C, and an S-lens RF level of 50. Resolution was set to 70,000 with an AGC target of 1e5.

## Results

### Sample preparation and glycan detection

In the past, the preparation of fluorescence-labeled N-glycans was a time-consuming procedure, usually employing overnight enzymatic N-glycan release and overnight labeling. Besides some kits promising rapid sample preparation, Aich et al. have recently introduced a rapid deglycosylation and labeling procedure which was used as a basis for further optimization in this work [19]. The aim of the optimized sample preparation was to efficiently reduce or remove byproducts of the reductive amination reaction, which might interfere with relative quantification of the glycans in the fluorescence trace. Dimethyl sulfoxide (DMSO) was identified as problematic, since purification via solid-phase extraction (SPE) was barely possible due to its high viscosity and high boiling point and the solubility of polar and non-polar compounds makes it a major source for impurities. DMSO was replaced with ethanol which was found to be an optimal solvent for the labeling reaction, because it can be obtained in high purity and it can be removed efficiently after the labeling reaction by evaporation. Furthermore, both the fluorophore tag anthranilic acid and the reductant 2-picoline borane are soluble in the used ethanol and acetic acid mixture. 2-Picoline borane was employed as a non-toxic alternative to the widely used sodium cyanoborohydride [18].

During optimization of sample preparation, it was ensured that sialic acids were preserved and that the investigated glycoproteins were fully deglycosylated. To achieve complete deglycosylation, complex glycosylated proteins other than IgGs required addition of a denaturing agent before incubation with PNGase F. Deglycosylation efficiency was determined using reducing capillary gel electrophoresis. Table 1 shows that for the tested molecules, three monoclonal antibodies (mAbs) and one complex glycosylated biopharmaceutical deglycosylation were complete.

**Table 1:** Efficiency of de-N-glycosylation of 100 μg glycoprotein assessed by reducing CE-SDS analysis. Percentage deglycosylation with standard deviation listed (*n* = 3). Deglycosylation can be considered as complete for all four reported molecules

	mAb1	mAb2	mAb3	Complex biopharmaceutical^1^
Control	2.4 ± 0.1%	0.2 ± 0.0%	0.6 ± 0.0%	3.5 ± 0.1%
PNGase F treated	99.1 ± 0.2%	99.4 ± 0.2%	99.6 ± 0.3%	98.6 ± 0.1%

^1^Protein was denatured with Rapigest prior to de-N-glycosylation

Using excitation and emission wavelength scans during chromatography, the optimal wavelengths were determined to provide the maximal signal-to-noise (S/N) ratio of 2-AA N-glycans. At an excitation wavelength of 350 nm and an emission wavelength of 440 nm, the highest S/N ratio was observed. The determined values from RPC and HILIC runs were identical, indicating that there was no influence from the mobile phase components.

### Selection of the stationary phase for optimal retention and selectivity of 2-AA N-glycans

**Fig. 1:**
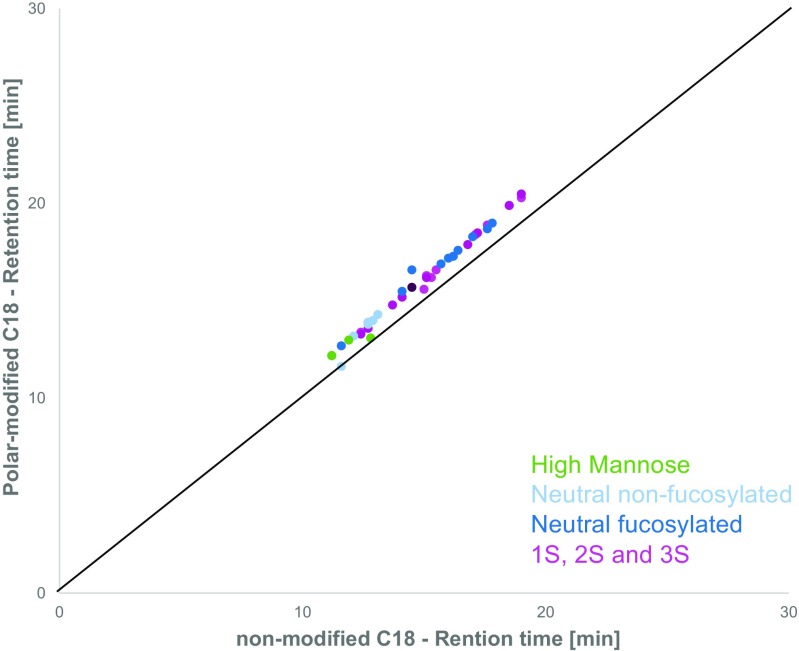
Representative scatter plot of retention times of 2-AA N-glycans on non-modified and polar-modified ODS phase. The black line indicates no difference in selectivity with an *r*^2^ of 1 and *s*^2^ of 0. N-Glycans are colored according to their type. On both stationary phases, all peaks elute between 10 and 20 min. Retention is slightly stretched towards the polar-modified packing, indicating a slightly improved selectivity (*s*^2^ = 0.01) on the polar-modified phase

**Fig. 2:**
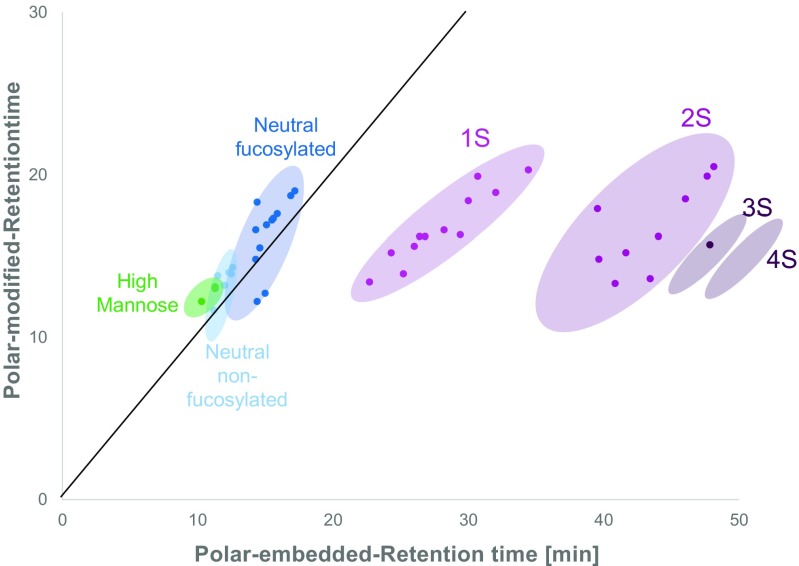
Representative scatter plot of retention times of 2-AA N-glycans on polar-modified and polar-embedded stationary phases, respectively. The black line indicates no difference in selectivity with an *r*^2^ of 1 and *s*^2^ of 0. N-Glycans are colored according to their type. Colored ellipses indicate the retention times of the different N-glycan groups. Retention of acidic N-glycans (1S, 2S, and 3S) is shifted towards the polar-embedded packing showing the improved selectivity (*s*^2^ = 0.78). The empty 4S ellipse indicates the retention time at which this type of N-glycans would elute since these N-glycans were only detected when employing polar-embedded reversed-phase chromatography

**Fig. 3:**
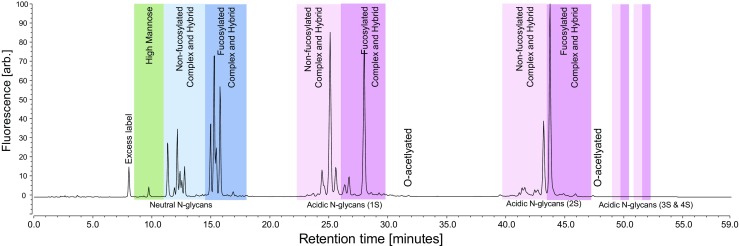
Example chromatogram of a complex glycosylated biopharmaceutical by UHP-RPC with fluorescence detection. Eluting peaks were identified by ESI-MS according to their exact mass and assigned to the different N-glycan types. Neutral N-glycans elute first with high mannose-type N-glycans eluting before non-fucosylated complex and hybrid N-glycans and fucosylated complex and hybrid N-glycans. Retention time increases with increasing numbers of terminal sialic acids. Within these groups, non-fucosylated N-glycans elute before fucosylated N-glycans followed by N-glycans with additional O-acetylation at the sialic acids

Different UHPLC columns with identical column dimensions (150 × 2.1 mm) and sub-2-μm particle size were tested representing three different reversed-phase technologies: one conventional ODS column without polar modifications, one ODS column with polar modifications on the particle surface, and one reversed-phase C14 column with polar-embedded amide functionality. Selectivity and peak capacity were used as evaluation criteria, since peak capacity is directly related to average resolution. Selectivity and peak capacity were determined as previously described by Neue (see Section 2.6) and are listed in Tables 2 and 3 [17, 20]. To compare the three columns, identical mobile phase composition, gradient steepness, and the same method run time were used, unless stated otherwise. A complex mixture of N-glycans containing up to four sialic acids was prepared to compare the stationary phases. The chromatography on the ODS column resulted in a chromatogram similar to previous findings where neutral N-glycans elute in groups beginning with high mannose, followed by non-fucosylated hybrid and complex and fucosylated hybrid and complex N-glycans. Neuraminic acid–carrying N-glycans eluted before the respective neutral variant. When analyzing complex N-glycan mixtures with a higher portion of sialic acids, chromatograms become crowded and separation into distinct groups is not possible, as illustrated in Fig. 1. The use of a polar-modified packings and C18 alkyl chains did not significantly improve the separation of the labeled N-glycans (Fig. 1). However, the stationary phase with the embedded polar group showed a drastic difference in overall selectivity. The difference in selectivity was determined to be *s*^2^ = 0.80 (see also Fig. 2). Figure 3 shows that the previously poorly retained acidic N-glycans were efficiently separated into distinct peak groups. With an increasing number of terminal sialic acids (1S, 2S, 3S, and 4S) the retention time increased. Within these groups, N-glycans are separated into non-fucose- and fucose-carrying groups with minimal overlap. From Fig. 3, it can be seen that the retention time window increases from approximately 10 min on the polar-modified ODS column to about 40 min on the column with polar-embedded amide functionality. For neutral N-glycans, selectivity is comparable between polar-embedded and polar-modified packings.

**Table 2:** Peak capacity of the analyzed molecules using the different columns (150 × 2.1 mm). Gradient time was 20 min for mAbs and 45 min for the complex glycosylated biopharmaceutical

	Non-modified	Polar-modified	Polar-embedded	Polar-embedded^1^
mAb1	60	72	81	79
mAb2	66	71	75	67
mAb3	n.a.	n.a.	83	77
Average mAb	63	72	80	74
Complex biopharmaceutical	51	57	157	147

^1^Mobile phase without alcohol modifiers

**Table 3:** Selectivity differences *s*^2^ for all compared columns and mobile phases

Column	Non-modified	Polar-modified	Polar-embedded	Polar-embedded^1^
Non-modified	–	0.01	–	0.80
Polar-modified	0.01	–	–	0.78
Polar-embedded	–	0.78	–	0.02
Polar-embedded^1^	0.80	0.80	0.02	–

^1^Mobile phase without alcohol modifiers

### Retention of 2-AA-labeled glycans on EPG phases

Overall chromatographic selectivity is dependent on an interplay of mobile phase composition, separation conditions, and the nature of the stationary phase [21]. Modern bonded phases are stable over a wide pH and temperature range, making the manipulation of temperature and pH some of the most important tools in a chromatographer’s toolkit [21]. To further our understanding of the retention mechanism, separation of 2-AA- and 2-AB-labeled N-glycans on the polar embedded phase was compared (see Electronic Supplementary Material (ESM) Fig. S1). The 2-AB-labeled N-glycans eluted earlier and selectivity among neutral N-glycans was lower compared to the 2-AA chromatogram. This shows that the 2-AA label is fundamental for the interaction with the stationary phase and that the sialic acids play an important role too. Changing the pH of the mobile phase primarily alters the ionization of ionogenic compounds, such as surface silanols and carboxylic acid groups of the analyte [22]. Increasing the pH of the mobile phase to 4.5 as used in HILIC chromatography with a 50 mM ammonium formate buffer greatly influenced the RP separation (ESM Fig. S2). At pH 4.5, the carboxylic acid group of 2-AA is deprotonated; thus, the labeled N-glycans were negatively charged. This led to mixed elution of neutral and acidic N-glycans and lower selectivity between neutral N-glycans. Using this pH 4.5 mobile phase in combination with 2-AB-conjugated N-glycans on the polar-embedded reversed phase further decreased the selectivity and resolution (ESM Fig. S3).

Protonation of the carboxylic acid groups of anthranilic acid was facilitated by low pH conditions to improve interaction with the bonded phase of both neuraminic acids and the conjugated fluorophore. It was observed that a mixture of 0.5% formic acid and 0.5% acetic acid in both mobile phases resulted in efficient and reproducible chromatographic results. The packing with embedded amide group interacted strongly with N-glycans with three and four terminal neuraminic acids (3S and 4S), so elution with only acetonitrile was barely possible.

**Fig. 4:**
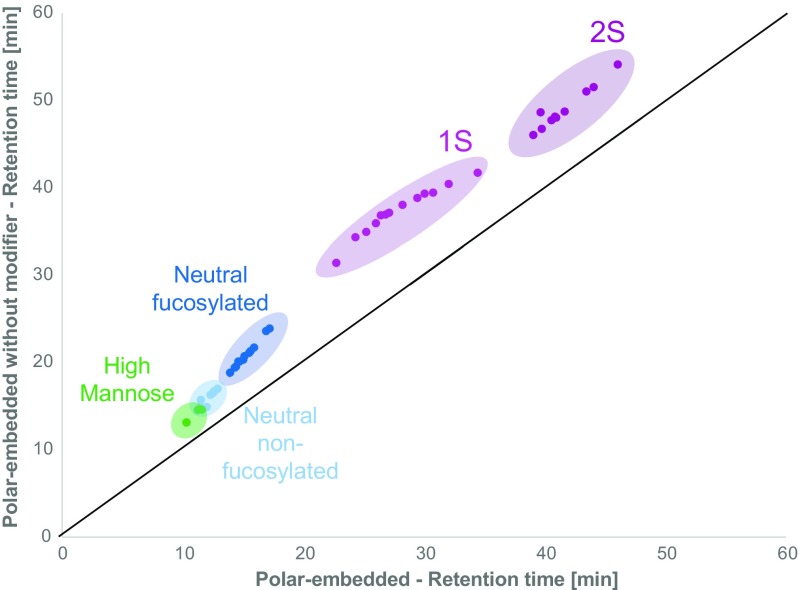
Influence of the mobile phase composition on the retention of 2-AA N-glycans on polar-embedded bonded phases. The black line indicates no difference in selectivity with an *r*^2^ of 1 and *s*^2^ of 0. N-Glycans are colored according to their type. Colored ellipses indicate the retention times of the different N-glycan types. Addition of modifiers (1-propanol and 1-butanol) enabled the elution of highly acidic species (3S and 4S) that where not eluted efficiently without the modifiers. In addition, peak capacity and selectivity (*s*^2^ = 0.02) are slightly higher using the modifiers

Multiple organic solvents were first preselected by running them under comparable chromatographic conditions to assess solvent strength and differences in selectivity. For that, methanol, ethanol, 1-propanol, 1-butanol, and acetonitrile were tested (ESM Table S1). Observations were in good agreement with expectations, showing increasing solvent strength in the order of methanol < ethanol < propanol < butanol. Co-elution of conjugated N-glycans with fluorescent reaction byproducts is a major concern in reversed-phase chromatography and thus included in the evaluation. The mobile phase was tuned to yield a suitable compromise of resolution, separate elution of by-products and N-glycans, and speed. The addition of aliphatic alcohols (i.e., n-propanol and 1-butanol) improved elution of 3S and 4S N-glycans while also slightly increasing selectivity, *s*^2^ = 0.02 (Fig. 4). Concentrations of 5% of 1-propanol and 1-butanol in the organic phase were found to be optimal and higher concentrations did not further improve selectivity or separation of impurities. Resulting peak capacities for the tested molecules and columns are listed in Table 2. Peak capacities increased from the conventional ODS packing to the polar-modified ODS packing, with the highest peak capacity values for the column with the embedded polar group as shown in Figs. 1, 2, and 4.

pH of mobile phases was tested with multiple mobile phase combinations. To yield reproducible separation of the distinct groups (neutral, S1, S2, S3, and S4), a low pH was necessary, as facilitated by 0.5% formic acid and 0.5% acetic acid.

### Application of method to IgG and complex glycosylated biopharmaceuticals

**Fig. 5:**
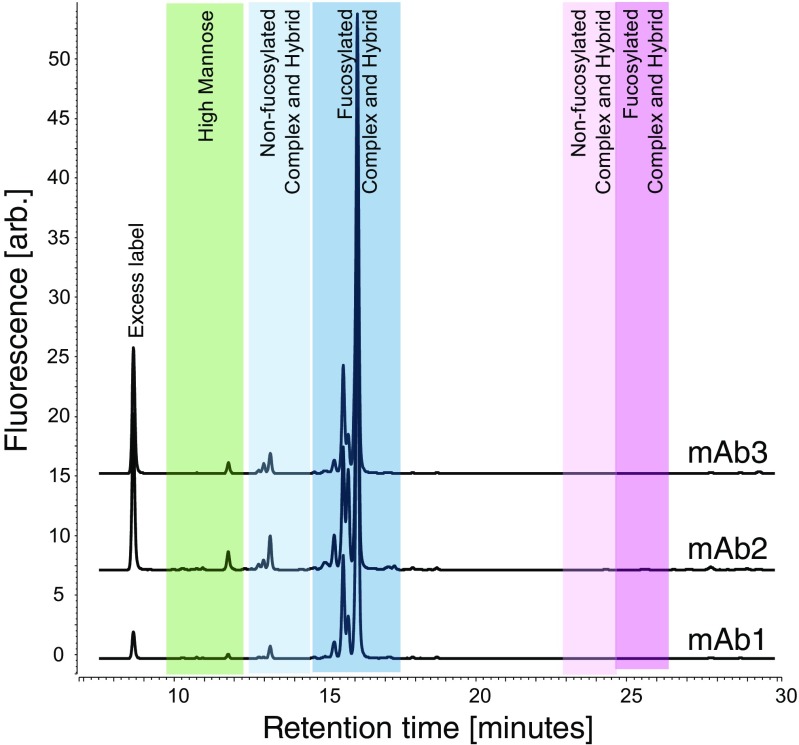
Chromatograms of the abbreviated gradient for the three tested mAbs. Eluting peaks were identified by ESI-MS according to their exact mass and assigned to the different N-glycan types. Elution order is identical to the one of the complex biopharmaceutical. The three mAbs contain different levels of high-mannose, non-fucosylated, and fucosylated N-glycans. Low levels of acidic N-glycans elute lastly

The developed analytical method was applied to characterize N-glycans of a complex glycosylated biopharmaceutical and three mAbs. Figure 3 shows a chromatogram of 2-AA N-glycans of the complex glycosylated biopharmaceutical. The different glycan groups are highlighted with colors. In addition to the previously described separation, additional types of N-glycans could be separated, acidic N-glycans containing O-acetylations at the terminal sialic acids. These modified N-glycans elute after their respective unmodified variants. More than 100 peaks were resolved, many of them with a relative abundance below 0.5%. Figure 5 illustrates the chromatograms obtained for three different mAbs. mAbs 1 and 2 have effector functionality (ADCC) which is influenced by non-fucosylated N-glycans eluting in a distinct group separated from their fucosylated counterparts allowing a prediction of ADCC activity from the glycan data. All mAbs contained low numbers and levels of acidic N-glycans which allowed a shorter and steeper gradient after the elution of the neutral N-glycans and a total runtime of only 30 min.

### Comparison of RPC to HILIC

**Fig. 6:**
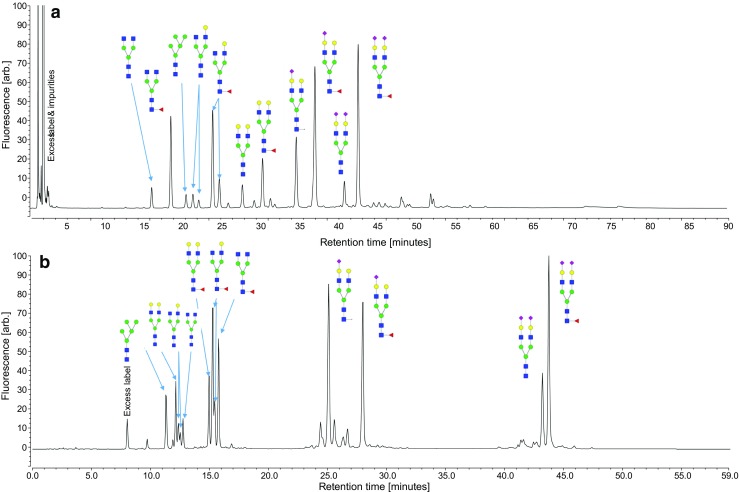
N-Glycans released from a complex glycosylated fusion protein separated with both (**a**) HILIC and (**b**) UHP-RPC with fluorescence detection. Main N-glycan structures are assigned to their respective peaks showing the differences in elution order between the two chromatography methods

The presented reversed-phase method was compared to the industry “gold-standard” HILIC. Figure 6 shows the chromatograms of a UHP-HILIC chromatogram (Fig. 6a) and the reversed-phase chromatogram (Fig. 6b). The most abundant peaks were labeled in both chromatograms demonstrating the selectivity differences. On the HILIC phase, 2-AA N-glycans elute according to their molecular size and hydrophilicity with smaller N-glycans eluting first. Notably, this results in co-elution of some fucosylated and non-fucosylated glycan species (ESM Fig. S6), which represents a major disadvantage, since it is essential to accurately quantify levels of afucosylated monoantennary glycans for assessing effector functions of IgGs. In contrast, separation via RPC allowed for the exact determination of these glycans, due to the separation into distinct groups, as shown in Fig. 5. Peaks in the RP chromatogram also were sharper; however, resolution between neutral N-gylcans was higher with HILIC. When coupled online to ESI-MS, the advantage of the acidic mobile phase used for RPC became obvious. Ionization was more efficient and higher MS signal intensities were obtained (ESM Figs. S4 and S5). Thus, overall MS sensitivity was greater when using RPC by approximately one order of magnitude.

## Discussion

As previously described, anthranilic acid is a suitable fluorophore label for efficient separation of N-glycans in reversed-phase chromatography. It was demonstrated that chromatography using polar-embedded amide functionality resulted in superior selectivity and resolution compared to previously published RPC methods. The main mechanism of retention is probably provided through interactions of the uncharged label and the N-glycan core structure with the alkyl chains of the stationary phase which was previously described to be higher for 2-AA N-glycans [13, 23]. The reason for the improved retention, based on the described results, is most likely due to electrostatic interactions of the polar neuraminic acids with the embedded amide group providing an additional mode of interaction with the polar-embedded bonded phase [24]. We show that both the hydrophobic label and pH are critical for retention in RPC and that the embedded polar groups in the alkyl chains represent an interaction site for sialic acids, since acidic N-glycan was efficiently separated on these phases, but neither on conventional ODS phases nor on ODS phases with polar surface modifications.

The addition of a more hydrophobic sugar like the deoxyhexose fucose led to a shift towards greater retention times, whereas an increasing number of hexose residues (galactose or mannose) leads to a decreased retention. At higher pH values, retention was greatly reduced for sialic acid–carrying glycans and separation into distinct groups based on type did not occur. RPC of labeled N-glycans provides an orthogonal separation mechanism to HILIC, resulting in the grouping of different types of N-glycans. Due to this separation into distinct groups, the presented method can lead to faster establishment of a structure-function relationship (e.g., ADCC and non-fucosylation), since the role in biological function is usually related to the N-glycan type [3]. Separation of the co-eluting N-glycans described in Fig. S6 (see ESM) with HILIC requires excessively long and shallow gradients with run times in the order of 150 min, which are not practical for routine applications [25]. Similar separation of sialic acid N-glycans can be achieved using weak anion exchange chromatography (WAX). However, WAX chromatography neither yields good separations of neutral N-glycans nor does it offer good compatibility with MS detection [26]. Its buffered mobile phases lead to low intensities in MS detection, even if negative ionization is applied. Reversed-phase chromatography is well understood and commonly applied in most analytical laboratories. Due to its favorable properties, such as the generally easy implementation, robustness, and compatibility with MS, the presented method represents an excellent alternative to the commonly applied HILIC, both in academic research and in the biopharmaceutical industry.

## Electronic supplementary material

ESM 1 (PDF 809 kb)

## Abbreviations

2-AA: Anthranilic acid
2-AB: 2-Aminobenzamide
ADCC: Antibody-dependent cellular cytotoxicity
CGE: Capillary gel electrophoresis
EPG: Embedded polar groups
HILIC: Hydrophilic interaction liquid chromatography
IgG: Immunoglobulin G
mAb: Monoclonal antibody
ODS: Octadecylsilane
PGC: Porous graphitized carbon
PNGase F: Peptide N-glycosidase F
RPC: Reversed-phase chromatography
SPE: Solid-phase extraction
