# Supplementary material for: High-selectivity profiling of released and labeled N-glycans via polar-embedded reversed-phase chromatography
Source: Anal Bioanal Chem. 2018 Nov 26;411(3):735–43. doi: 10.1007/s00216-018-1495-7 (PMC6338698; doi:10.1007/s00216-018-1495-7)
Supplement: Supplementary file 1 — (PDF 809 kb) [file 216_2018_1495_MOESM1_ESM.pdf]

Analytical and Bioanalytical Chemistry

Electronic Supplementary Material

High-selectivity profiling of released and labeled N-glycans via polar-embedded reversed-phase chromatography

Johannes G. Wilhelm, Marco Dehling, Fabian Higel

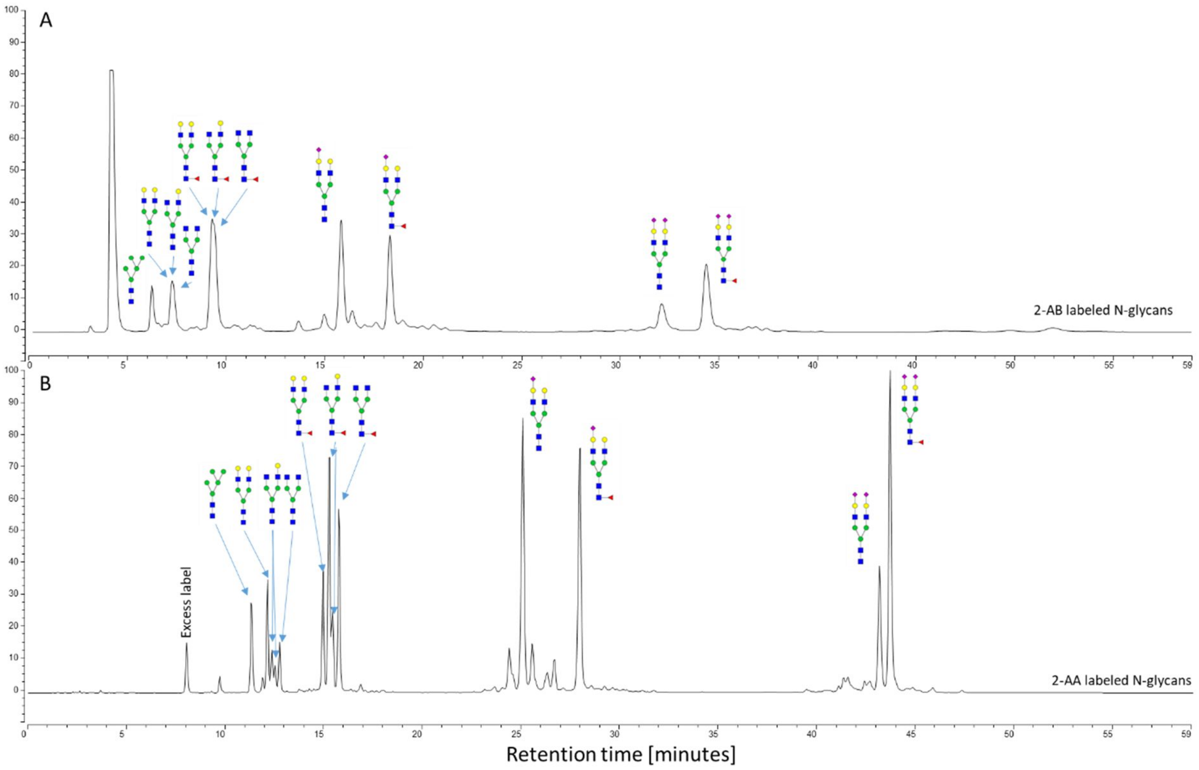

Fig. S1 Comparison of reversed phase chromatograms of 2-AB (A) and 2-AA (B) labeled N-glycans of a complex biopharmaceutical. 2-AB N-glycans elute earlier and with a lower resolution and broader peak shape. Increasing number of terminal sialic acids improves the retention of 2-AB labeled N-glycans

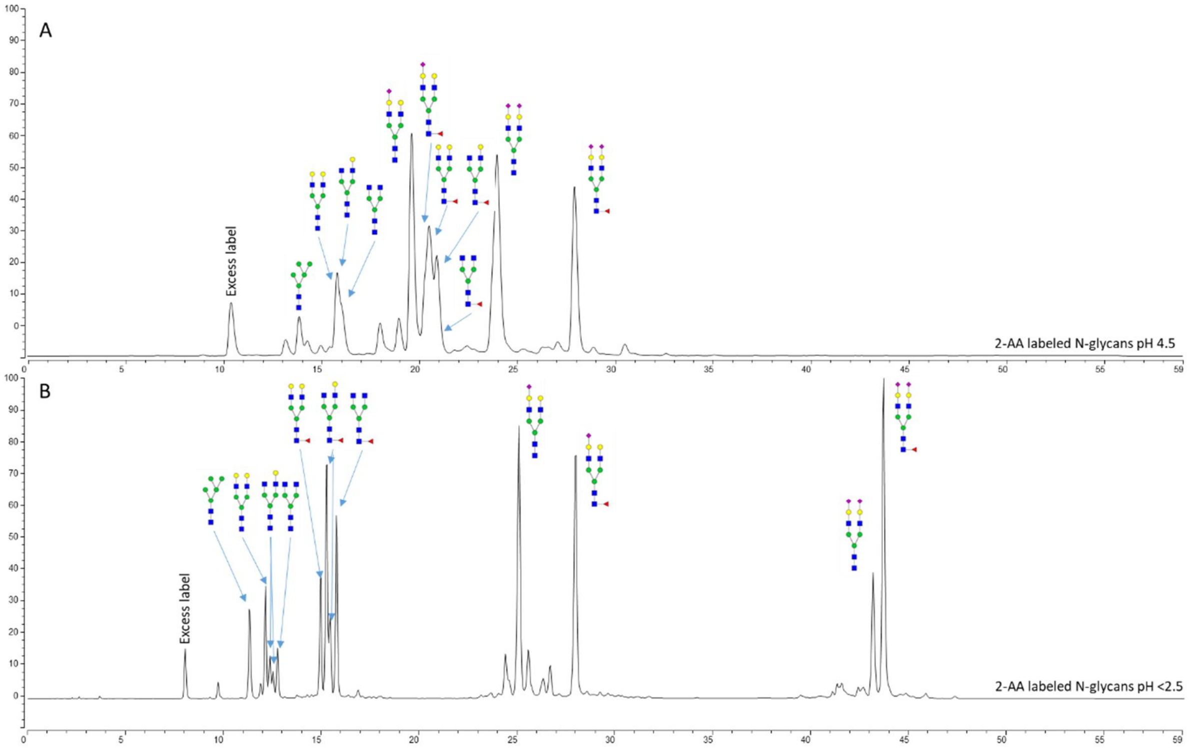

Fig. S2 Influence of pH on separation of 2-AA labeled N-glycans on reversed phase. (A) At a mobile phase pH of 4.5 the anthranilic acid label is deprotonated. Labeled N-glycans elute earlier with mixed elution of different N-glycan types (acidic and neutral). (B) The mobile phase with a pH < 2.5 results in the separation of the labeled N-glycans according to their type

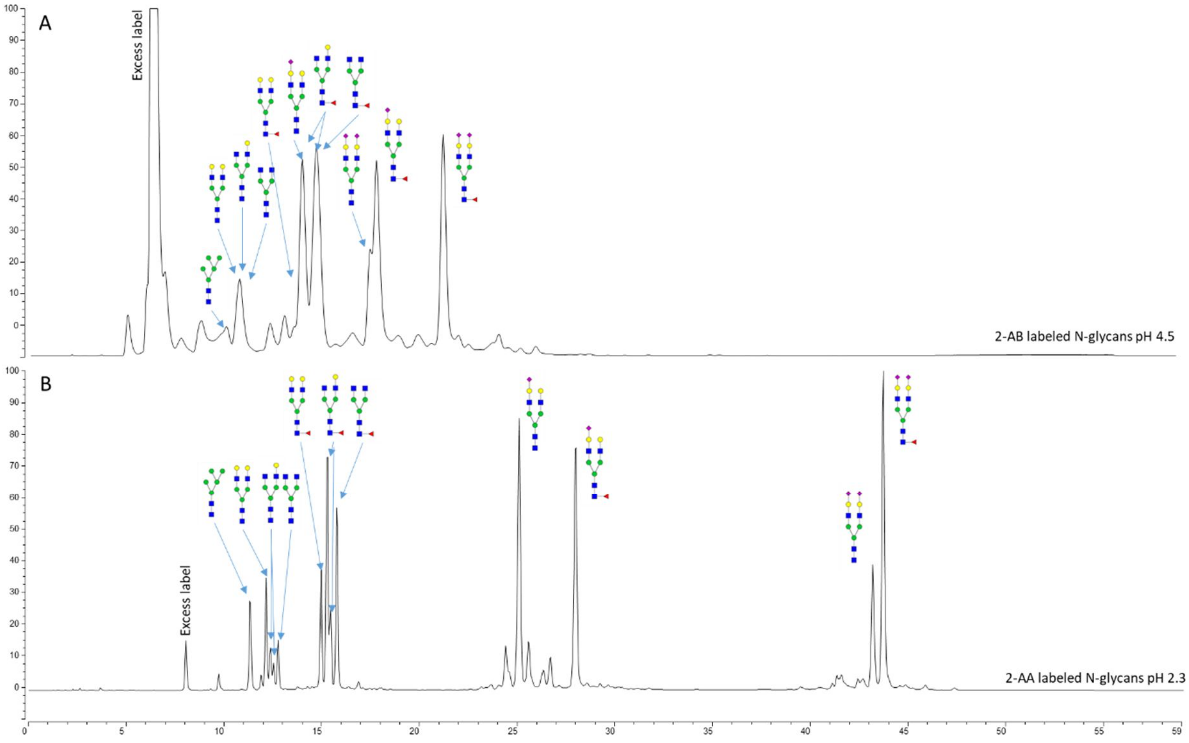

Fig. S3 Influence of pH on separation of 2-AB and 2-AA labeled N-glycans on reversed phase. (A). Labeled 2-AB N-glycans elute early with mixed elution of different N-glycan types (acidic and neutral). (B) The mobile phase with a pH < 2.5 results in the separation of the 2-AA labeled N-glycans according to their type

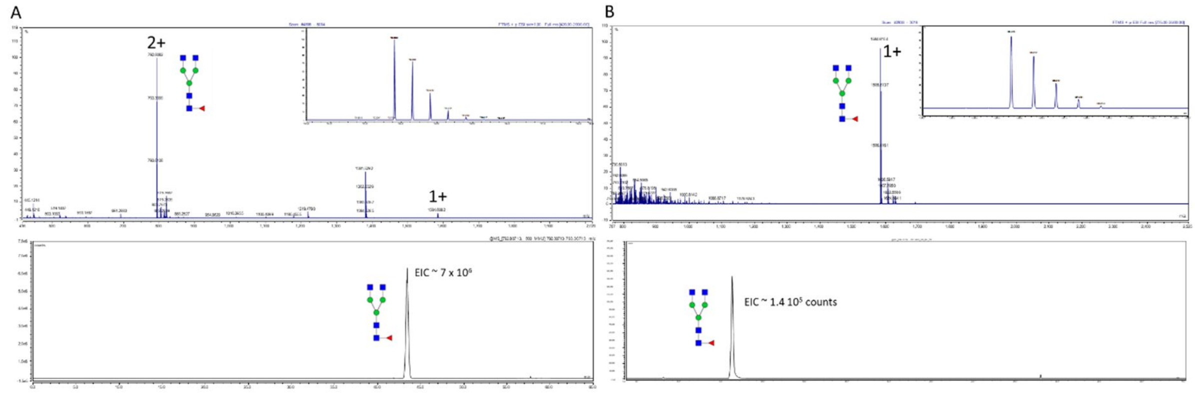

Fig. S4 MS ionization differences of 2-AA labeled complex type N-glycans from RPC and HILIC mobile phases. UHPLC was coupled on-line to the ESI source. A) 2-AA labeled N-glycans ionize as singly and doubly charged ions from RPC mobile phase. Intensity of the corresponding extracted ion chromatogram is 7.6 x 10^6 counts. B) 2-AA labeled N-glycans ionize as singly charged ions from HILIC mobile phases. Intensity of the corresponding extracted ion chromatogram is 1.4 x 10^5 counts and one order of magnitude lower

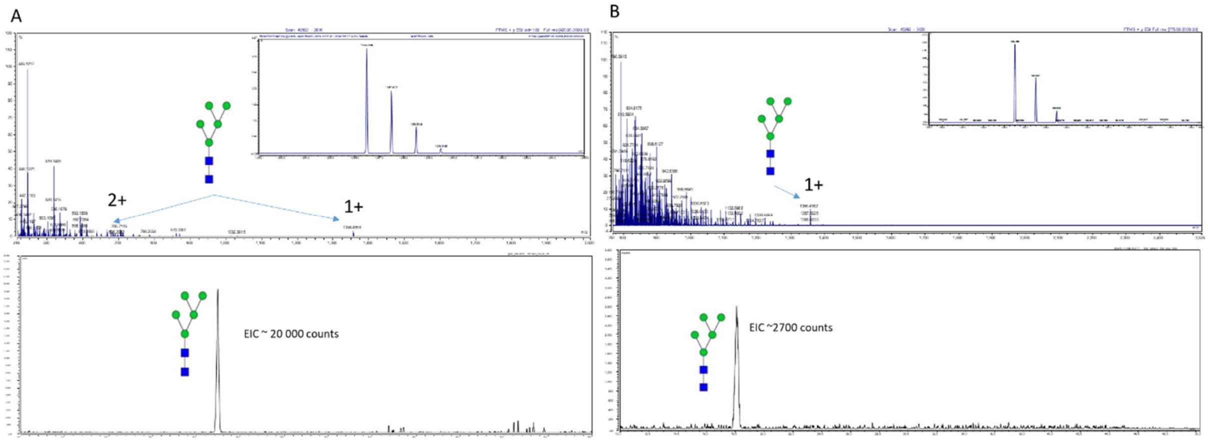

Fig. S5 MS ionization differences of 2-AA labeled high mannose type N-glycans from RPC and HILIC mobile phases. UHPLC was coupled on-line to the ESI source. A) 2-AA labeled N-glycans ionize as singly and doubly charged ions from RPC mobile phase. Intensity of the corresponding extracted ion chromatogram is 20,000 counts. B) 2-AA labeled N-glycans ionize as singly charged ions from HILIC mobile phases. Intensity of the corresponding extracted ion chromatogram is 2700 counts and one order of magnitude lower

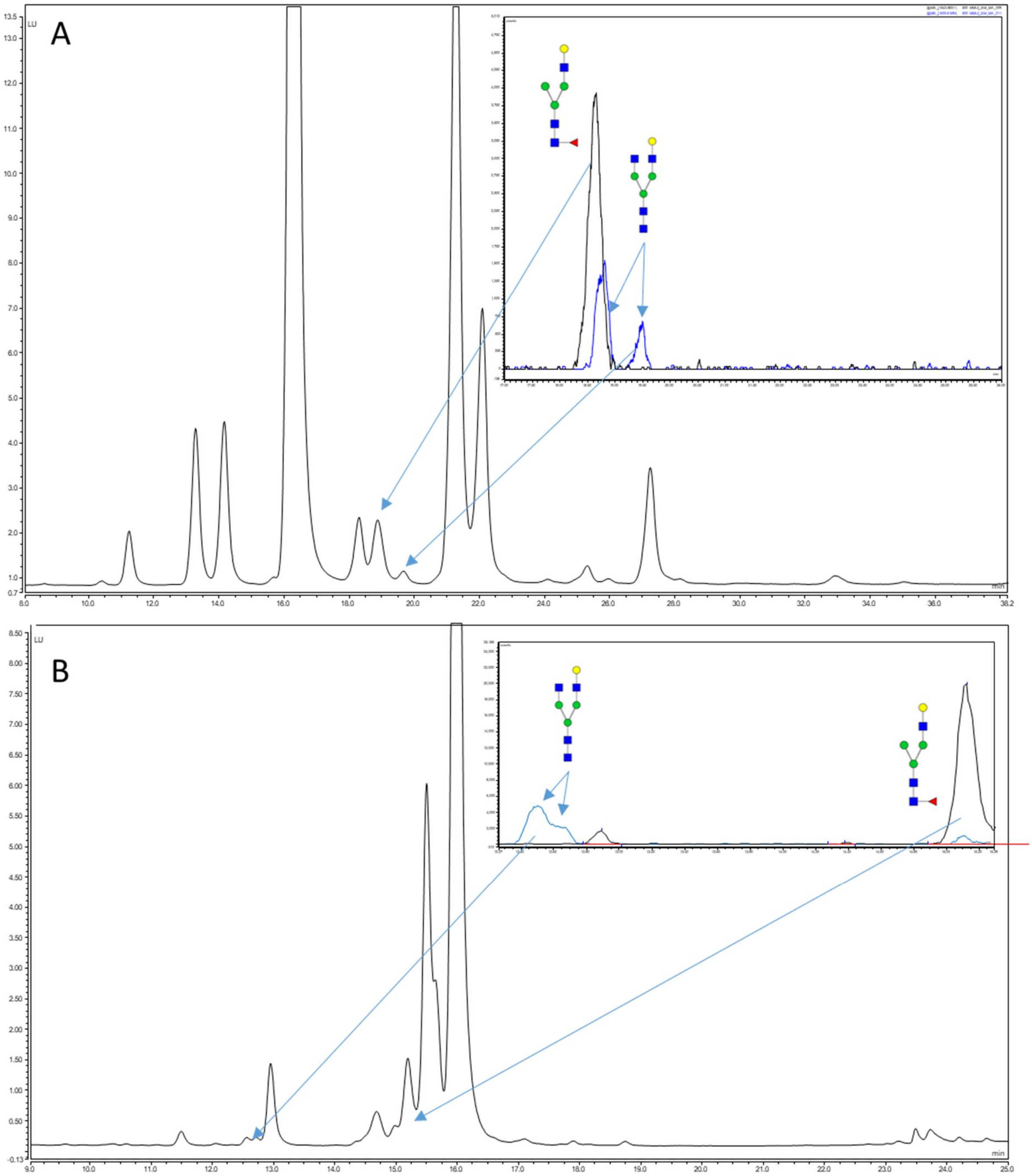

Fig S6 Separation of 2-AA labeled N-glycans on HILIC phases (A) leads to co-elution of fucosylated and non-fucosylated N-glycans. Separation of labeled N-glycans on reversed phase (B) allows for the exacted determination of fucosylation levels

Table S1 Tested organic solvents and modifiers and their influence on the retention of 2-AA labeled N-glycans on reversed phases

Organic modifier	Effect
Methanol	Low solvent strength and low selectivity, several byproducts coeluted with major glycans
Ethanol	Medium solvent strength, comparable to acetonitrile, albeit lower selectivity
1-Propanol	High solvent strength, high selectivity for neutral glycans observed
1-Butanol	Exceptionally high solvent strength, high selectivity for neutral glycans observed
Acetonitrile	Medium solvent strength, improved elution of sialic acid-carrying glycans
